# Evaluation of agricultural and rural pollution under environmental measures in the Yangtze River Economic Belt, China

**DOI:** 10.1038/s41598-023-42837-0

**Published:** 2023-09-19

**Authors:** Aiping Pang, Dawei Wang

**Affiliations:** 1 Department of Public Management, Party School of Nanjing Municipal Committee of CPC (Nanjing Academy of Administration), Nanjing, 210046 China; 2 North China Municipal Engineering Design and Research Institute Co., LTD., Tianjin, 300074 China

**Keywords:** Environmental impact, Sustainability

## Abstract

In this study, material flow and spatial analysis methods were used to evaluate and predict the spatial–temporal pattern evolution of agricultural and rural nitrogen (N) flow in the Yangtze River Economic Belt in China from 1949 to 2050 and to analyze agricultural and rural pollution control by environmental measures. The results showed that since the founding of the People’s Republic of China, the crop harvest in the Yangtze River Economic Belt has shown an overall upward trend, and the increase in the period from 1979 to 1997 was the fastest, with an average annual increase rate of 3.8%. Since the reform and opening up, N loss (storage) increased from 50.97 × 10^8^ kgN in 1978 to 140.15 × 10^8^ kgN in 2014, a 2.75-fold increase. In 2015, China began to implement measures to prevent and control agricultural and rural pollution, and N loss (storage) decreased yearly. In 2019, the N loss (storage) decreased by 18.22% compared with that in 2015, but it was still high. Each year, 113.44 × 10^8^ kgN was still lost to the atmosphere, water and soil, which was 1.53 times the amount of N harvested with crops. The N loss rate was as high as 60%. Before 2014, N discharge into surface water from agricultural and rural areas in the Yangtze River Economic Belt increased annually, especially after 1978, with an average growth rate of 4.76%, leading to severe nonpoint source pollution. With the implementation of the pollution control policy, the N lost to surface water began to show a downward trend in 2015, but it was still 2.17 times higher than the environmental risk threshold in 2019. According to the prediction, under the scenarios of the business-as-usual, fertilizer reduction, engineering and rural improvement patterns, the N emissions from the system to surface water in 2050 are expected to be reduced by 25.76%, 45.5%, 30% and 30%, respectively, compared with those in 2019, but will still be higher than the environmental risk threshold. Under the integrated pattern, the N emissions to surface water are reduced to 4.32 × 10^8^ kgN in 2050, which is lower than the environmental risk threshold and can achieve the goal of nonpoint source pollution control. A single environmental measure cannot effectively control nonpoint source pollution. It is necessary to promote an integrated pattern to achieve green and sustainable development of agriculture in the Yangtze River Economic Belt.

The most frequent nitrogen (N)-related activities occur in agricultural and rural areas, and N is directly related to food security, crop productivity, environmental effects and human health^1,2^. The pollution caused by agricultural production and rural life is generally assessed using the N and phosphorus (P) input into the environment as the evaluation index. Excessive N use is the main cause of water quality deterioration, water ecosystem degradation and biodiversity reduction^3^, while a lack of N threatens food security^4^. Quantitative evaluation of the N cycle in agricultural production and rural life is the main means to trace and control agricultural and rural pollution. The most frequently used methods include N budget balance and material flow analysis. By introducing the concept of “process” into the N balance calculation, the material flow analysis method can not only quantify different N flow paths but also quantitatively evaluate the internal relationships of different N flows^5–8^. However, the flow of N from social systems into natural systems is not only restricted by land cover but also affected by meteorological and geographical factors such as terrain, soil type, temperature, and rainfall. Therefore, the emission risk varies greatly in different regions^9^. The development of 3S technology (Remote Sensing, Global Positioning System and Geographic Information System) promoted research on the spatial distribution pattern of N in different regions, allowing managers to regulate high-risk areas in a more targeted way. The 14th Five-Year Plan period of China is a period of in-depth progress in the prevention and control of nonpoint source pollution. It is also a period of critical challenges in the environmental protection and green development of the Yangtze River Economic Belt^10^. In this study, material flow analysis combined with a spatial analysis model was used to evaluate the spatial and temporal distribution of N cycles in agricultural and rural areas from 1949 to 2050. Then, combined with environmental measures, we further evaluate and predict the influence of environmental measures on the prevention and control of agricultural pollution, especially nonpoint source pollution in the Yangtze River Economic Belt.

## Study area and current environmental policy

The Yangtze River Economic Belt covers 11 provinces (cities), including Shanghai, Jiangsu, Zhejiang, Anhui, Jiangxi, Hubei, Hunan, Chongqing, Sichuan, Yunnan and Guizhou, and covers 21% of China’s total land area. It is an important area for grain, oil, livestock, poultry and aquatic products. Because the economy and population are relatively concentrated and the river system is very developed, the Yangtze River Economic Belt is the area with the most serious nonpoint source pollution problem in China^10^. In recent years, the government of China has implemented a series of agricultural and rural pollution control programs: (1) the Ministry of Agriculture issued the “Implementation Opinions on Fighting the Battle against Non-Point Source Pollution” and the “Action Plan for Zero Growth of Fertilizer Use by 2020” in April 2015, which put forward the “One control, two reduction and three basic goals” policy that includes reducing the amount of fertilizer application and implementing the action of zero growth of fertilizer; (2) since 2015, the State Council, the Ministry of Agriculture, the National Development and Reform Commission and other departments have issued a series of documents on the resource utilization of excrement and straw, which put forward measures such as comprehensive utilization of livestock and poultry excrement, returning straw to the field, optimization of breeding layout and development of moderate-scale breeding, to comprehensively promote the recycling of livestock and poultry breeding wastes; (3) the State Council, the Ministry of Housing and Urban‒Rural Development, the Ministry of Ecology and Environment, the National Development and Reform Commission and the Ministry of Agriculture issued a series of plans on the treatment of rural sewage and garbage, which put forward measures to promote the construction of rural sewage treatment facilities and improve the collection and treatment of household garbage. All provinces and cities along the Yangtze River Economic Belt have carried out comprehensive pollution control in agriculture and rural areas, effectively reducing pollution caused by agricultural production, rural residents living and animal breeding. However, agricultural pollution, especially nonpoint sources, is still the main pollution in the Yangtze River due to the high resource development intensity and numerous historical debts^11^. Controlling agricultural and rural pollution while ensuring food security is important to improve the water quality and promote the high-quality development of the Yangtze River Economic Belt.

## Methods

**Figure 1:**
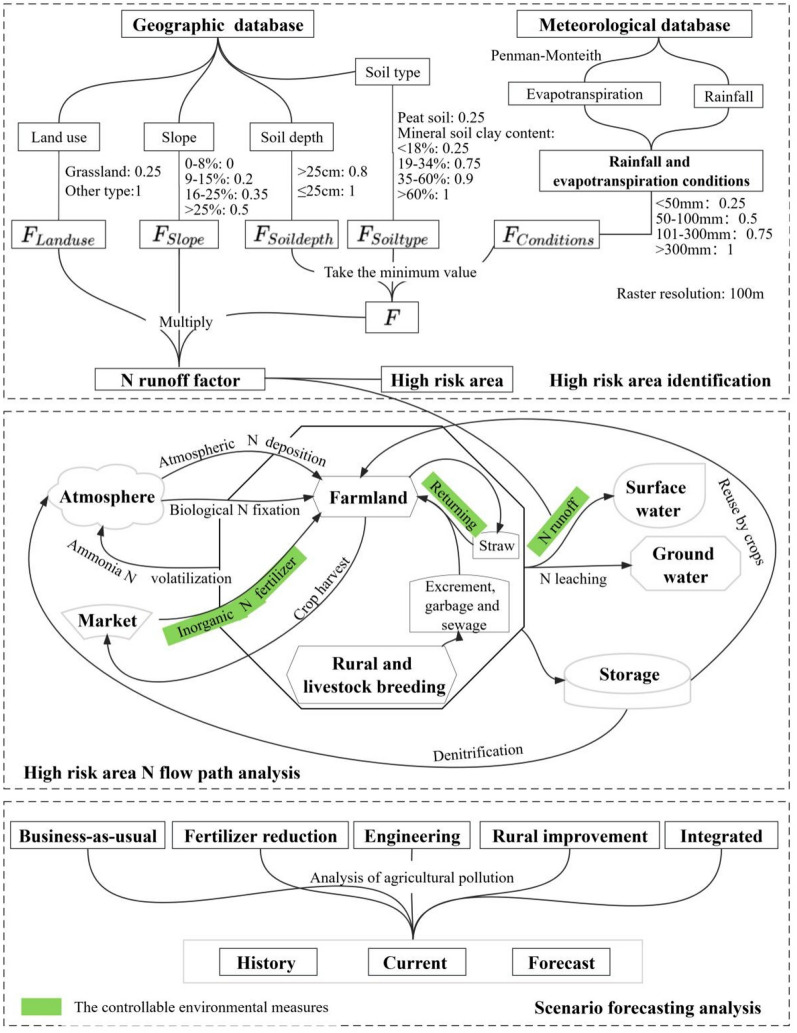
Research framework.

This study is divided into three modules (Fig. 1): the first step is to obtain basic geographic data through field investigation, online collection, purchase/negotiation by geographic information departments, etc., and identify high-risk pollution areas based on 3S technology; the second step is to measure the N flow pathways in agricultural and rural areas, as well as the N exchange with the environment; and the third step is to evaluate and predict the influence of different measures on pollution control by scenario analysis.

### High-risk area identification

N runoff loss refers to the process in which N migrates out of soil and into surface water along with runoff during rainfall. In the spatialization scheme, this study assumed that each pixel (100 m resolution) is a relatively independent unit. Environmental-related factors determine how much N surplus will stay in the pixel and how much N surplus will be transferred from this pixel to other subsystems. The spatialization of N loss factors can reveal the high-risk areas of N loss in the Yangtze River Economic Belt. If the environmental-related conditions meet the N loss needs, part of the N surplus could be washed away to surface water in the form of N runoff; otherwise, the N will stay or leach into ground water. This study built a spatial model to determine the N runoff factor based on the EU-27^9^ method and the geographic and meteorological database. All geographic and meteorological data were converted to raster form and then unified to 100 m resolution per pixel on the ArcGIS platform.

### N flow path analysis

In this study, material flow analysis was used to evaluate the N cycle in agricultural and rural areas of the Yangtze River Economic Belt. The input flow includes atmospheric N deposition, biological N fixation and inorganic N fertilizer. Internal flows include excrement and garbage returning, straw returning, and rural N surplus (untreated excrement, garbage, and sewage); the output flows include crop harvest, ammonia N volatilization, N runoff, N leaching, N storage, denitrification and other N flow paths. The calculation method of each N flow is shown in Table 1. The N flows of inorganic N fertilizer, the returning of excrement, garbage and straw, and N runoff are the controllable factor in the Scenario forecasting analysis.

**Table 1:** N flow pathways.

Type	N flow	Calculation formula	Parameter meaning
Input N flows	Atmospheric N deposition	${\mathrm{F}}_{\mathrm{atmospheric \,deposition}}={\uplambda }_{\mathrm{atmospheric \,deposition}}{\mathrm{A}}_{\mathrm{cultivated\, land}}$	${\mathrm{F}}_{\mathrm{atmospheric\, deposition}}$ is the atmospheric N deposition, in kgN/a; ${\uplambda }_{\mathrm{atmospheric \,deposition}}$ is the atmospheric N deposition coefficients, in kgN·ha^−1^·a^−1^; and ${\mathrm{A}}_{\mathrm{cultivated\, land}}$ is the cultivation area, in ha
	Biological N fixation	${\mathrm{F}}_{\mathrm{biological \,N \,fixation}}={\uplambda }_{\mathrm{ legume}}{\mathrm{A}}_{\mathrm{ legume}}+{\uplambda }_{\mathrm{paddy}}{\mathrm{A}}_{\mathrm{paddy}}+{\uplambda }_{\mathrm{dryland \,crop}}{\sum }_{\mathrm{i}=1}^{\mathrm{n}}{({\mathrm{A}}_{\mathrm{dryland }})}_{\mathrm{i}}$	${\mathrm{F}}_{\mathrm{biological \,N\,fixation}}$ is the amount of N entering the system through biological N fixation, in kgN·a^−1^; ${\uplambda }_{\mathrm{ legume}}$, ${\uplambda }_{\mathrm{paddy}}$ and ${\uplambda }_{\mathrm{dryland\, crop}}$ are the N fixation coefficients of legumes, rice and dryland crops, in kgN·ha^−1^·a^−1^; and ${\mathrm{A}}_{\mathrm{ legume}}$, ${\mathrm{A}}_{\mathrm{paddy}}$ and ${\mathrm{A}}_{\mathrm{dryland}}$ are the planting areas of legumes, rice and dryland crops, respectively, in ha
	Inorganic N fertilizer	${\mathrm{F}}_{\mathrm{inorganic}}={\mathrm{P}}_{\mathrm{inorganic}}+{\uplambda }_{\mathrm{compound }}{\mathrm{P}}_{\mathrm{compound}}$	${\mathrm{F}}_{\mathrm{inorganic}}$ is the amount of N input into the system by inorganic N fertilizer application, in kgN·a^−1^; ${\uplambda }_{\mathrm{compound}}$ is N conversion in compound fertilizer, dimensionless; and ${\mathrm{P}}_{\mathrm{inorganic}}$ and ${\mathrm{P}}_{\mathrm{compound}}$ are the application amounts of inorganic N fertilizer and compound fertilizer, respectively, in kg·a^−1^
Internal N flows	Organic N fertilizer (excrement and garbage return)	${\mathrm{F}}_{\mathrm{organic\, N\, fertilizer }}={\uplambda }_{\mathrm{return}}{\mathrm{F}}_{\mathrm{excrement \,and\, garbage}}$ ${\mathrm{F}}_{\mathrm{excrement \,and\, garbage}}=365\left[({{\uplambda }_{\mathrm{excrement\,P}}{\upmu }_{\mathrm{excrement\,P}}+{\uplambda }_{\mathrm{garbage}}{\upmu }_{\mathrm{garbage}})\mathrm{P}}_{\mathrm{population}}+{\sum }_{\mathrm{i}=1}^{\mathrm{n}}{{(\uplambda }_{\mathrm{excrement \,A}}{\upmu }_{\mathrm{excrement\, A}}{\mathrm{P}}_{\mathrm{animal}})}_{\mathrm{i}}\right]$ $${\mathrm{P}}_{\mathrm{animal}}=\left\{\begin{array}{cc}{\mathrm{P}}_{\mathrm{slaughtered}}& {\mathrm{day}}_{\mathrm{breeding \,cycle}}\ge 365\mathrm{d}\\ {(\mathrm{P}}_{\mathrm{slaughtered}}+{\mathrm{P}}_{\mathrm{breeding}-\mathrm{stock}})/(\frac{365}{{\mathrm{day}}_{\mathrm{breeding\, cycle}}}+1)& {\mathrm{day}}_{\mathrm{breeding \,cycle}}<365\mathrm{d}\end{array}\right.$$	${\mathrm{F}}_{\mathrm{organic \,N \,fertilizer}}$ is the amount of N that enters the system through excrement and garbage recycle pathways, in kgN·a^−1^; ${\uplambda }_{\mathrm{return}}$ is the return coefficients of excrement and household garbage, dimensionless;${\mathrm{F}}_{\mathrm{excrement\, and \,garbage}}$ is the amount of N produced by excrement and household garbage, in kgN·a^−1^; ${\uplambda }_{\mathrm{excrement\,P}}$,${\uplambda }_{\mathrm{excrement \,A}}$ and ${\uplambda }_{\mathrm{garbage}}$ are the N content coefficients of rural resident, livestock and poultry excrement and household garbage, dimensionless; ${\upmu }_{\mathrm{excrement\,P}}$, ${\upmu }_{\mathrm{excrement \,A}}$ and ${\upmu }_{\mathrm{garbage}}$ are the daily production of rural resident, livestock and poultry excrement and household garbage, in kg·person^−1^·day^−1^; ${\mathrm{P}}_{\mathrm{population}}$ is the population of rural residents in person·a^−1^; ${\mathrm{P}}_{\mathrm{animal}}$ is the daily number of breeding animals, in capita·a^-1^; ${\mathrm{P}}_{\mathrm{slaughtered}}$ and ${\mathrm{P}}_{\mathrm{breeding}-\mathrm{stock}}$ are breeding-stock and slaughtered livestock and poultry, in capita·a^−1^; and ${\mathrm{day}}_{\mathrm{breeding\, cycle}}$ is the breeding cycle, in days
	Straw return	${\mathrm{F}}_{\mathrm{straw\, return}}={\uplambda }_{\mathrm{straw}}{\sum }_{\mathrm{i}=1}^{\mathrm{n}}{{(\upmu }_{\mathrm{straw}}\uptheta {\mathrm{P}}_{\mathrm{grain}})}_{\mathrm{i}}$	${\mathrm{F}}_{\mathrm{straw\, return}}$ is the amount of N that enters the system through straw return, in kgN·a^−1^; ${\uplambda }_{\mathrm{straw}}$ is the straw return rate to the field, dimensionless;${\upmu }_{\mathrm{straw}}$ is the N content in straw, dimensionless; $\theta$ is the ratio of grain to grass, dimensionless; ${\mathrm{P}}_{\mathrm{grain}}$ is the annual crop yield, in kg·a^-1^; and i represents the crop type, including rice, wheat, sorghum, bean, sugar cane, peanut, beet, corn, potato, cotton, vegetables, and fruits, dimensionless
	Rural N surplus (untreated excrement, garbage and sewage)	${\mathrm{F}}_{\mathrm{N \,surplus \,R}}=(1-{\uplambda }_{\mathrm{return}}){\mathrm{F}}_{\mathrm{manure \,and\, garbage}}+365[{({\uplambda }_{\mathrm{sewage}}{\upmu }_{\mathrm{sewage}})\mathrm{P}}_{\mathrm{population}}$	${\mathrm{F}}_{\mathrm{N \,surplus\, R}}$ is N content in untreated excrement, garbage and sewage in rural areas, in kgN·a^−1^; ${\uplambda }_{\mathrm{sewage}}$ is sewage discharge coefficient of rural residents, in kg·person^−1^·d^−1^; ${\upmu }_{\mathrm{sewage}}$ is N content in sewage, in kgN·kg^−1^
Output N flows	Crop harvest	${\mathrm{F}}_{\mathrm{crop\, harvest}}=\sum_{\mathrm{i}=1}^{\mathrm{n}}{({\upmu }_{\mathrm{grain}}{\mathrm{P}}_{\mathrm{grain}})}_{\mathrm{i}}$	${\mathrm{F}}_{\mathrm{crop \,harvest}}$ is the amount of N output through the crop harvest, in kgN·a^−1^; ${\upmu }_{\mathrm{grain}}$ is the N content in the available parts of crops (grains, vegetables and fruits), dimensionless; ${\mathrm{P}}_{\mathrm{grain}}$ is the annual crop yield, in kg·a^−1^; and i represents the crop type, dimensionless
	Ammonia N volatilization	${\mathrm{F}}_{\mathrm{volatilization}}={\uplambda }_{\mathrm{ volatilization}}{\mathrm{F}}_{\mathrm{inorganic}}+{\upgamma }_{\mathrm{volatilization}}{\uplambda }_{\mathrm{return}}{\mathrm{F}}_{\mathrm{excrement \,and\, garbage}}+{\updelta }_{\mathrm{volatilization}}(1-{\uplambda }_{\mathrm{return}}){\mathrm{F}}_{\mathrm{excrement and garbage}}$	${\mathrm{F}}_{\mathrm{volatilization}}$ is the ammonia N volatilization, in kgN·a^−1^; ${\uplambda }_{\mathrm{ volatilization}}$, ${\upgamma }_{\mathrm{volatilization}}$ and ${\updelta }_{\mathrm{volatilization}}$ are the ammonia volatilization coefficients of inorganic N fertilizer, organic N fertilizer, and untreated excrement and garbage, dimensionless
	N runoff	${\mathrm{F}}_{\mathrm{runoff}}={\uplambda }_{\mathrm{runoff}}({\mathrm{F}}_{\mathrm{N surples F}}+{\mathrm{F}}_{\mathrm{N \,surplus \,R}})$ ${\mathrm{F}}_{\mathrm{N\, surplus\, F}}={\mathrm{F}}_{\mathrm{atmospheric deposition}}+{\mathrm{F}}_{\mathrm{biological \,N \,fixation}}+{\mathrm{F}}_{\mathrm{inorganic}}+{\mathrm{F}}_{\mathrm{organic\, N\, fertilizer }}+{\mathrm{F}}_{\mathrm{straw\, return}}-{\mathrm{F}}_{\mathrm{crop \,harvest}}-{\mathrm{F}}_{\mathrm{volatilization}}$	${\mathrm{F}}_{\mathrm{runoff}}$ is the amount of N lost through N runoff, in kgN·a^−1^; ${\uplambda }_{\mathrm{runoff}}$ is the N runoff coefficient, dimensionless; ${\mathrm{F}}_{\mathrm{N \,surplus\, F}}$ represents the N surplus in the planting area, in kgN·a^−1^
	N leaching	${\mathrm{F}}_{\mathrm{leaching}}={\uplambda }_{\mathrm{leaching}}({\mathrm{F}}_{\mathrm{N surples F}}+{\mathrm{F}}_{\mathrm{N \,surplus\, R}}-{\mathrm{F}}_{\mathrm{runoff}})$	${\mathrm{F}}_{\mathrm{leaching}}$ is the amount of N output from the system through N leaching, in kgN·a^−1^; ${\uplambda }_{\mathrm{leaching}}$ is the N leaching coefficient, dimensionless
	N storage	${\mathrm{F}}_{\mathrm{other}}={\mathrm{F}}_{\mathrm{N\, surples \,F}}+{\mathrm{F}}_{\mathrm{N \,surplus \,R}}-{\mathrm{F}}_{\mathrm{runoff}}-{\mathrm{F}}_{\mathrm{leaching}}$	The excess N stored in the soil, which may enter the atmosphere through denitrification or may be taken up by crops

### Scenario forecasting analysis

The settings of activity and environmental measure-related parameters during 2020–2050 are shown in Table 2.

**Table 2:** Parameter settings for scenario analysis.

Parameters and patterns		Business-as- usual	Fertilizer reduction	Engineering^1^	Rural improvement	Integrated
Activity parameter	Cultivated land	According to the Land Administration Law of China, the conversion of cultivated land into noncultivated land is strictly controlled, so the amount of cultivated land remains unchanged (in 2019), totaling 44.93 million hectares				
	Population	Projected by data from 1949 to 2019, the forecast is 160.79, 74.33 and 14.15 million in 2030, 2040 and 2050				
	Planting area	Projected by data from 1949 to 2019, the forecast is 66.065, 66.221 and 66.492 million hectares in 2030, 2040 and 2050				
	Daily number of breeding animals	Projected by data from 1949 to 2019, the average daily breeding capacity of large livestock (cattle, horses, donkeys and sheep) is 95.52, 99.35 and 104.43 million capital in 2030, 2040 and 2050, respectively. The average daily breeding capacity of pigs is 121.19, 75.76 and 49.04 million, respectively. The average daily breeding capacity of poultry is 998.47, 1080.96 and 1167.21 million, respectively				
	Crop harvest	Projected by data from 1949 to 2019, the total output of rice, wheat, cotton, sorghum, soybean, sugarcane, peanut, sugar beet, corn, potato, vegetables and fruits is 790.27, 932.22 and 1079.13 million tons in 2030, 2040 and 2050, respectively				
Environmental measure-related parameter	Inorganic N fertilizer	Unchanged in 2019	Gradual decrease by 30% by 2050	Unchanged in 2019	Unchanged in 2019	Gradual decrease by 30% by 2050
	Disposal of excrement, sewage, straw and garbage		Unchanged in 2019		The return rate of excrement, straw and garbage to the field remains unchanged in 2019, and the untreated part was made into fertilizer or feed. Sewage goes to the sewage treatment plant. Zero N emissions is achieved	
	Engineering			Gradual decrease by 10% by 2050	Unchanged in 2019	Gradual decrease by 10% by 2050

^1^Engineering measures mainly reduce N runoff via construction of ecological ditches, constructed wetlands, ecological filters and other buffers in areas with a high risk of nonpoint source pollution.

### Environmental risk threshold

Since the reform and opening up of China in 1978, agricultural environmental pollution in the Yangtze River Economic Belt has gradually increased and shown a worsening trend under the comprehensive influence of the rapid growth of chemical fertilizer consumption, continuous increase in animal husbandry production, and gradual increase in untreated garbage and sewage from rural residents. Therefore, in the forecasting scenarios, the N loss in 1979 was taken as the environmental risk threshold. If the predicted data were higher than this threshold, it indicated that some regions of the Yangtze River Economic Belt might face pollution risks.

### Data collection

(1) Basic data on population size, inorganic N fertilizer amount, cultivation area, crop yield, breeding-stock and slaughtered livestock and poultry, and crop planting area were mainly obtained from the Statistical Yearbook of Shanghai, Anhui, Jiangsu, Hubei, Zhejiang, Chongqing, Jiangxi, Hunan, Sichuan, Yunnan and Guizhou (http://www.stats.gov.cn/). Missing data were supplemented by China’s economic and social research platform (https://data.cnki.net/NewHome/index). (2) N-related coefficients, including N fixation coefficients, atmospheric N deposition coefficient, and daily production and N content coefficients of rural resident, livestock and poultry excrement and household garbage, ratio of grain to grass, straw return rate, N content in the straw and available parts of crops, excrement return rate, and ammonia N volatilization rate, among others, were mainly obtained from the literature^12–24^. (3) The spatial data used to calculate the N runoff coefficient and N leaching coefficient, such as land use type, soil type, elevation and rainfall, were mainly obtained from online databases (http://www.globallandcover.com/GLC30Download/index.aspx, http://westdc.westgis.ac.cn, http://www.jspacesystems and http://data.cma.gov.cn). Spatial data were processed by interpolation, transformation, clipping and uniform resolution (100 m) on the ArcGIS platform.

### Ethical statement

In our study, the studies of involving plants (rice, wheat, sorghum, bean, sugar cane, peanuts, beets, corn, potatoes, cotton, vegetables, and fruits) has been carried out in accordance with relevant institutional, national, and international guidelines and legislation.

## Results and discussion

### N flow pathways and high-risk areas

**Figure 2:**
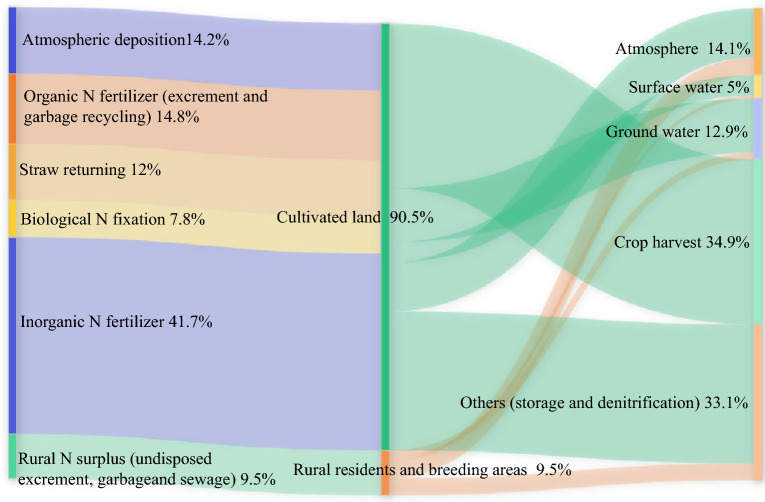
Proportion of N flow pathways in the Yangtze River Economic Belt (2019).

The main N flow pathways and their proportions in agricultural and rural areas are shown in Fig. 2. A total of 90.5% of the N input in the system is in cultivated land. The proportion of N in untreated excrement, garbage and sewage in rural areas is relatively low at 9.5% but much of it is lost directly to the environment. The proportion of inorganic N fertilizer was the highest (41.7%), and the proportions of excrement and garbage recycling, atmospheric deposition, straw return and biological N fixation were 14.8%, 14.2%, 12% and 7.8%, respectively. A total of 65.1% of N is lost or temporarily stored in the environment in various forms, and the remaining 34.9% leaves the system in the form of crop harvesting. Additionally, 14.1% of N entered the atmosphere in the form of ammonia volatilization, including organic and inorganic fertilizers in farmland, excrement piled up randomly by rural residents, discarded garbage and directly discharged sewage. The N in the system input into groundwater and surface water accounted for 12.9% and 5%, respectively. The former entered groundwater through leaching and lateral seepage, and the latter entered surface water bodies such as rivers, lakes and reservoirs through runoff processes. It is worth noting that the N runoff process, which accounts for only 5% of the total loss in the system, is the main cause of nonpoint source pollution in the Yangtze River Economic Belt. A total of 33.1% of N is temporarily stored in the soil, and part of it is reduced to gaseous nitride and N gas and released back to the atmosphere by microorganisms under anaerobic conditions. This process may not only pollute the atmosphere but also cause a great waste of N.

**Figure 3:**
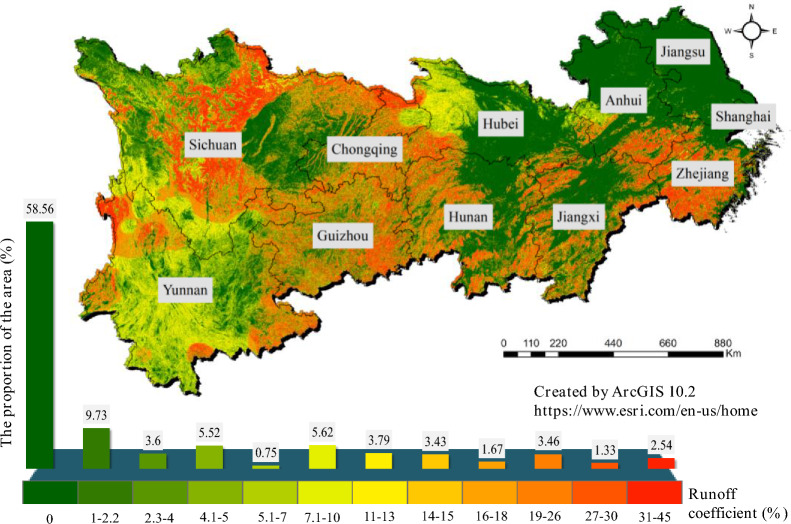
Risk area and its proportion of non-point source pollution in the Yangtze River Economic Belt (2019).

Surface runoff generated by rainfall will cause N in soil to migrate along with surface runoff, resulting in N loss. The amount of N loss depends not only on the level of N in soil but also on regional soil type, rainfall, topography, surface vegetation and human management measures. Figure 3 shows the risk area and its proportion of nonpoint source pollution in the Yangtze River Economic Belt in 2019. The N runoff coefficient of the Yangtze River Economic Belt ranges from 0 to 45%. A total of 58.56% of the area does not have the conditions for runoff, where excess N in the soil is lost to the environment by other means or stored in the soil and later taken up by crops. The area with N runoff coefficients between 4.1% and 10% accounts for 11.89% of the total area of the Yangtze River Economic Belt, and the area with N runoff coefficients between 7.1% and 10% accounts for 13.68%. The area with N runoff coefficients greater than 30% accounts for 2.54% of the total area of the Yangtze River Economic Belt, and 31% to 45% of the N surplus in this area will be directly lost to the surrounding surface water. For areas with a high risk of N runoff, only reducing the use of N fertilizer and increasing the recycling rate of excrement are not enough to reduce the N runoff. Engineering measures such as buffer zones and fallow sites should be further applied to reduce the amount of N lost to surface water.

### N input and crop harvest

**Figure 4:**
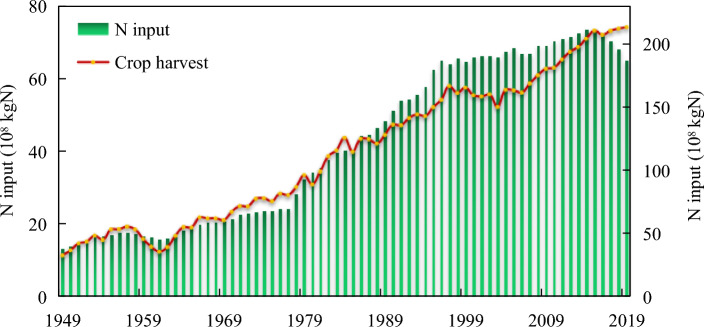
N input and crop harvest in the Yangtze River Economic Belt.

Food security is important for national stability. The Yangtze River Economic Belt is an important agricultural region in China, covering the production of grain, cotton, oil and other bulk agricultural products. Figure 4 shows the N input (including inorganic N fertilizer, organic N fertilizer such as excrement, straw and garbage returned to the field, atmospheric N deposition and biological N fixation) and the N harvested with crops (including grain crops, vegetables and fruits) during 1949–2019. Since the founding of the People’s Republic of China, the crop harvest in the Yangtze River Economic Belt has shown an overall upward trend, from 11.29 × 10^8^ kgN in 1949 to 74.18 × 10^8^ kgN in 2019, a 6.6-fold increase. Under the influence of N fertilizer input and agricultural policy, different time periods showed different characteristics. From 1949 to 1978, N input was generally low and was significantly affected by natural disasters. However, the crop harvest still showed a small upward trend increasing by 1.67 times in 30 years. In 1978, China’s rural management system was relaxed, greatly fostering enthusiasm among farmers to produce. At the same time, the introduction of inorganic N fertilizer greatly increased the N input. The 1979–1997 period was the period with the fastest growth in agricultural production. Crop harvest increased from 33.5 × 10^8^ kgN in 1979 to 58.03 × 10^8^ kgN in 1997, for an increase of 73.23%. From 1998 to 2003, affected by the adjustment of agricultural structure and the relatively low farmer income, the crop harvest in the Yangtze River Economic Belt continued to decrease, with a decrease of 10%. After 2004, with the implementation of agricultural tax exemption and subsidy policies, crop yield in the Yangtze River Economic Belt began to increase significantly, from 56.96 × 10^8^ kgN in 2004 to 73.24 × 10^8^ kgN in 2015, for an increase of 28.59%. Due to the restriction of agricultural and rural environmental policies, N input decreased significantly in 2015, from 211.22 × 10^8^ kgN in 2015 to 187.62 × 10^8^ kgN in 2019, for a decrease of 11.13%. Crop harvest in the Yangtze River Economic Belt still maintained an increasing trend, but the increase rate was significantly lower than that during 2004–2015 due to the decrease in total N input.

**Figure 5:**
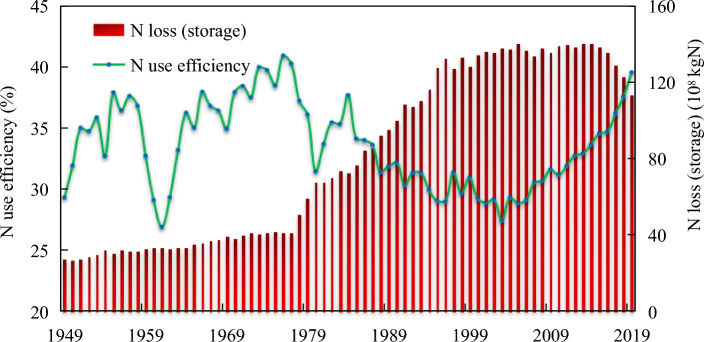
N use efficiency and N loss (storage) in the Yangtze River Economic Belt.

Figure 5 shows the N use efficiency and N loss (storage) in the Yangtze River Economic Belt. Since the reform and opening up of China in 1978, N input into the system has been increasing, while the crop growth potential is limited. Part of N is lost to the environment or stored in the soil, and it is subsequently absorbed and utilized by crops or further lost through denitrification, runoff and leaching^17^. N loss (storage) increased 2.75 times from 50.97 × 10^8^ kgN in 1978 to 140.15 × 10^8^ kgN in 2014. In 2015, the Chinese government launched measures for the prevention and control of nonpoint source pollution, and since then, N loss (storage) in the Yangtze River Economic Belt has shown a decreasing yearly trend. In 2019, N loss was 18.22% lower than that in 2015, but it was still high. Every year, 113.44 × 10^8^ kgN N was lost to the atmosphere, water bodies and soil, which was 1.53 times the N harvested with crops in the same year. The N loss rate was as high as 60%.

The average N use efficiency in the Yangtze River Economic Belt was 33.6% over the years analyzed. Under the traditional farming mode of organic fertilizer use before 1978, the N use efficiency was relatively high but fluctuated significantly with environmental factors. From 1949 to 1978, the N utilization rate ranged from 26.89 to 40.91%. Then, with the increase in inorganic N input, excess N flowed into the environment or was stored in the soil while supporting crop production. During 1979–2003, N use efficiency showed a downward trend, from 36.07% in 1979 to 27.42% in 2003. Benefiting from the improvement in agricultural planting efficiency and quality, N use efficiency in the Yangtze River Economic Belt began to show a rising trend, from 29.3% in 2005 to 39.54% in 2019.

### History, present status and prediction of agricultural nonpoint source pollution

**Figure 6:**
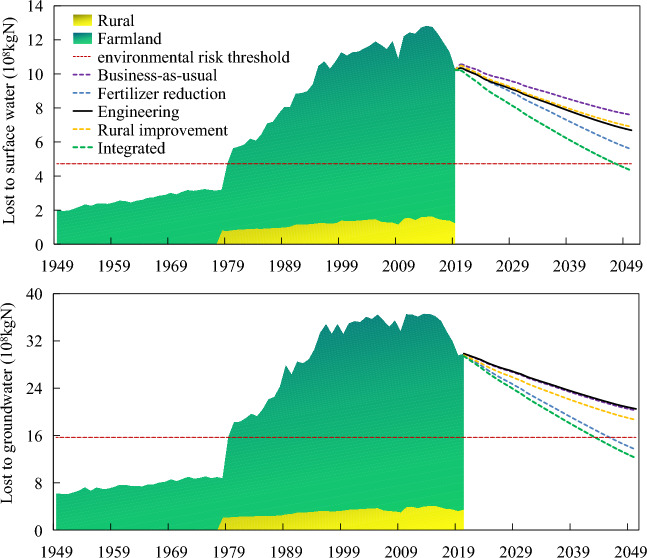
Current situation and prediction of N loss in the Yangtze River Economic Belt.

Figure 6 shows the current situation and prediction of N loss to surface and groundwater. During 1978–2014, the N lost to surface water showed an increasing yearly trend, with an average annual increase of 0.23 × 10^8^ kgN, from 4.72 × 10^8^ kgN in 1979 to 12.81 × 10^8^ kgN in 2014. After the implementation of the agricultural nonpoint source pollution control policy, the N entering surface water began to show a downward trend after 2015, but it was still at a high level of 10.22 × 10^8^ kgN in 2019, 2.17 times higher than the environmental risk threshold. Based on the average level from 1949 to 2019, the N discharged from cultivated land to surface water is 8.35 times that from rural areas, which is the main area where N runoff occurs. However, from the perspective of the material flow cycle, N-containing materials such as excrement and garbage in rural areas cannot enter cultivated land in the form of organic fertilizer, which will lead to increased N emissions in rural areas. At the same time, to ensure food production, farmers will choose to add more inorganic N fertilizer in cultivation, which will also cause N loss and waste. In addition, compared with the vast farmland, the rural area is smaller and the unit area of N emission is higher, so there is still a risk of nonpoint source pollution at the regional scale.

According to the scenario prediction, the business-as-usual pattern can promote a continuous decline in N emissions from the system to the surface water environment. The N emissions from the system to surface water in 2050 are expected to be 7.59 × 10^8^ kgN, which is 25.76% lower than that in 2019. Under the fertilizer reduction pattern, the inorganic N application rate in 2050 was reduced by 30% compared with that in 2019, and the N emissions from the system to surface water were significantly reduced to 5.57 × 10^8^ kgN, 45.5% less than that in 2019. Under the engineering and rural improvement pattern, N emissions to surface water showed a similar downward trend, and the emission reduction effect was between that of the business-as-usual pattern and the fertilizer reduction pattern, with a relative reduction of approximately 30% compared to 2019. However, after the implementation of the above four patterns, N discharge to surface water will still be higher than the environmental risk threshold. Under the integrated pattern, the N emissions to surface water are reduced to 4.32 × 10^8^ kgN in 2050, which is lower than the environmental risk threshold. In the Yangtze River Economic Belt, initial results have been achieved in controlling nonpoint source agricultural pollution, but the implementation of a single measure cannot reach the goal of completely controlling nonpoint source pollution. It is necessary to continue to promote fertilizer reduction, excrement and straw return and rural sewage treatment measures and to take engineering measures such as building buffer zones in high-risk areas.

The excess N discharge to groundwater in the Yangtze River Economic Belt system has a similar pattern to that to surface water. From 1978 to 2014, emissions showed a trend of substantial increase and began to decline with the implementation of pollution control measures but were still at a high level. In the scenario prediction, the implementation of different measures has different effects on emission reduction. The engineering pattern has little impact on the N discharge to groundwater, so the discharge trend is similar to that of the business-as-usual pattern. Both the fertilizer reduction pattern and integrated pattern could reduce systemic N emissions to groundwater below environmental risk thresholds before 2050.

## Conclusions and policy implications

In 2006, the Chinese government listed the control of agricultural source pollution and the reduction of chemical fertilizer application into the Outline of the 11th Five-Year Plan for National Economic and Social Development and then adopted a series of measures, including “One control, two reduction and three basic measures”, which have achieved initial results. In 2019, the Yangtze River Basin eliminated inferior Class V water bodies for the first time. N application and loss have shown an incremental turning point, and N discharge into surface water in 2019 decreased by 19.74% compared with 2015, but it is still at a high level. Agricultural pollution in the Yangtze River Economic Belt is still severe. By depicting the temporal and spatial patterns of agricultural and rural N in the Yangtze River Economic Belt and analyzing the impact of agricultural and rural environmental measures on nonpoint source pollution, we obtained the following conclusions.

1. The Yangtze River Economic Belt faces the dual goals of achieving food security and pollution control. Since the founding of the People’s Republic of China, crop harvest in the Yangtze River Economic Belt has shown an overall upward trend. Affected by N input and agricultural policies, different time periods present different characteristics. During 2015–2019, N input continued to decline, crop harvest increased less, and N loss showed a downward trend in this period. In 2019, N loss decreased by 18.22% compared with that in 2015, but 113.44 × 10^8^ kgN of N was still lost to the atmosphere, water and soil every year. The N loss was 1.53 times the amount of N harvested with crops, and the N loss rate was as high as 60%. During the “14th Five-Year Plan” period, agricultural environmental policies need to meet the dual objectives of food security and agricultural nonpoint source pollution control. Therefore, while further reducing the amount and intensity of chemical fertilizer, attention should also be paid to the application of organic fertilizer and the improvement of N use efficiency.
2. The risk of nonpoint source pollution varies greatly among different systems and regions in the Yangtze River Economic Belt. The average N runoff in rural residential areas accounted for only 12.27% of the total N loss from 1949 to 2019. As measures such as the construction of sewage treatment facilities in rural areas lag behind the reduction of agricultural N fertilizer, the proportion of N runoff is expected to rise to 15.05% by 2050, which may lead to local water pollution. Therefore, it is suggested that rural areas take measures such as the construction of sewage treatment plants and garbage recycling systems. There are considerable regional differences in agricultural pollution. Typically, 58.56% of the area does not have runoff conditions. The area with a high risk of nonpoint source pollution accounts for 2.54% of the total area, and 31–45% of the N surplus in this area will be directly lost to the surrounding surface water. Agricultural nonpoint source control and management measures should be promoted according to local conditions.
3. The implementation of the integrated pattern will ensure effective control of nonpoint source pollution in the Yangtze River Economic Belt. From the forecast scenario, after the implementation of the business-as-usual, fertilizer reduction, engineering and rural improvement patterns, the N emissions to surface water were still higher than the environmental risk threshold. Only under the integrated pattern can N emissions fall below the environmental risk threshold. The control of agricultural nonpoint source pollution in the Yangtze River Economic Belt has achieved some results, but the implementation of a single measure cannot achieve the goal of agricultural pollution control. Fertilizer reduction, excrement and straw recycling, and rural sewage treatment need to be sustained. At the same time, it is necessary to construct ecological ditches, artificial wetlands, ecological filters and other buffers to reduce N runoff in high-risk areas.

## Data Availability

The datasets used and/or analyzed during the current study are available from the corresponding author on reasonable request.
